# Iodide of Sodium vs. Iodide of Potassium

**Published:** 1886-10

**Authors:** 


IODIDE OF SODIUM VERSUS IODIDE OF POTASSIUM.—A recent leading article in the *British Medical Journal* thus sums up the advantages of iodide of sodium over iodide of potassium: "(1) It can be used therapeutically for almost all, certainly the chief, purposes for which potassium iodide is used, and with similar beneficial results. (2) Sodium iodide is more assimilable than the iodide of potassium, both locally to the digestive organs and to the general system. (3) That as a result many of the local and general undesirable effects which are produced by potassium iodide do not follow the use of sodium iodide."

